# Left Atrial Strain as a Predictor of Left Ventricular Diastolic Dysfunction in Patients with Arterial Hypertension

**DOI:** 10.3390/medicina58020156

**Published:** 2022-01-20

**Authors:** Tatjana Miljković, Aleksandra Ilić, Aleksandra Milovančev, Marija Bjelobrk, Maja Stefanović, Anastazija Stojšić-Milosavljević, Snežana Tadić, Miodrag Golubović, Tanja Popov, Milovan Petrović

**Affiliations:** 1 Faculty of Medicine, University of Novi Sad, 21000 Novi Sad, Serbia; tatjana.miljkovic@mf.uns.ac.rs (T.M.); aleksandra.ilic@mf.uns.ac.rs (A.I.); marija.bjelobrk@mf.uns.ac.rs (M.B.); maja.stefanovic@mf.uns.ac.rs (M.S.); anastazija.stojsic@mf.uns.ac.rs (A.S.-M.); snezana.tadic@mf.uns.ac.rs (S.T.); miodrag.golubovic@mf.uns.ac.rs (M.G.); tanja.popov@mf.uns.ac.rs (T.P.); milovan.petrovic@mf.uns.ac.rs (M.P.); 2 Institute of Cardiovascular Diseases of Vojvodina, 21208 Sremska Kamenica, Serbia

**Keywords:** arterial hypertension, echocardiography, diastolic dysfunction, strain, left atrium

## Abstract

*Background and Objectives:* There is emerging evidence of the usefulness of left atrial strain (LAS) in the assessment of diastolic dysfunction (DD). In this study we assess the sensitivity and specificity of LAS, to determine cut-off values and their association to DD with increased left atrial pressure (LAP) in patients with well-treated arterial hypertension. *Materials and Methods:* We performed a cross-sectional study on 180 subjects with well-treated arterial hypertension. All patients underwent transthoracic echocardiography. Patients were divided into two groups: a group without increased LAP and/or DD and a group with increased LAP DD. *Results:* In multivariate logistic regression, LAS proved to be the strongest statistically significant predictor of DD with increased LAP (OR 0.834, *p* < 0.0005), with AUC 0.885 and a set cut-off value of 24.27% with high sensitivity of 78.9% and specificity of 84.6%. The set cut-off for LAS > 24.27% was significantly highly prevalent in the group of DD with increased LAP 78.9% when compared to the group without increased LAP 15.4%, *p* < 0.0001. *Conclusion:* The findings of this study suggest that LAS could be a useful and highly sensitive and specific marker in the evaluation of DD. There is the potential for using LAS in everyday practice as a standard parameter in diastolic function assessment.

## 1. Introduction

Early detection of asymptomatic diastolic dysfunction (DD) is crucial in preventing incident heart failure and improving the survival of patients with arterial hypertension [1]. The first guidelines for the evaluation of DD that included a wide range of 10 traditionally used diastolic parameters with several diagnostic algorithms were released in 2009 [2]. In 2016, new guidelines that are currently being used [3] excluded several previously validated parameters and simplified the assessment of diastolic function with a restructured, stepped approach and focus on four key variables [4]. Even with this simplified approach, some individuals’ DD remains indeterminate, with no clear message on what to do in such cases. There is a constant need for seeking new highly sensitive and specific parameters in the detection of DD, especially with elevated left atrial pressure (LAP) as it represents a risk factor for the progression to heart failure with preserved ejection fraction (HFPEF) [5]. Left atrial strain (LAS) is emerging as a significant index of left atrial (LA) dysfunction [1] and an early marker of DD when common echocardiographic parameters are still normal [6]. Hence, we wanted to investigate its potential role as a single predictor of DD. This study aimed to assess the sensitivity and specificity of peak LAS, to determine cut-off values and their association to DD with increased LAP in patients with well-treated arterial hypertension.

## 2. Materials and Methods

### 2.1. Patient Selection

This cross-sectional study, approved by the institutional review protocol/ethical committee, included 180 patients of both sexes, in whom arterial hypertension had been previously diagnosed and treated. All participants underwent a 24-h ambulatory blood pressure monitoring (ABPM) and transthoracic echocardiographic examination. Inclusion criteria were normal values of arterial blood pressure during ABPM, sinus rhythm in electrocardiogram (ECG), left ventricular (LV) ejection fraction (EF) ≥ 55%, no significant valvular heart disease (defined as moderate or severe regurgitation or stenosis) and no associated cardiac diseases, previous cardiac valve or revascularization surgery or secondary/gestational arterial hypertension. Based on the echocardiography exam, according to the 2016 American Society of Echocardiography (ASE) guidelines [3], all patients were classified into two groups: first group, 104 (57.77%) patients with no DD or with DD without elevated LAP-grade I DD. Second group, 76 (42.22%) patients with DD with elevated LAP–grade II or higher DD. Patients were treated with one or more of the following classes of antihypertensive drugs: angiotensin-converting enzyme (ACE) inhibitors, angiotensin receptor blockers (ARBs), calcium channel blockers (CCBs), diuretics and ß- blockers.

### 2.2. Blood Pressure Measurement

Blood pressure (BP) measurements were performed, according to the 2018 recommendation of the Task Force for the management of arterial hypertension of the European Society of Hypertension (ESH) and the European Society of Cardiology (ESC) [7], during the 24-h recording period using the Cardio Tens device (Meditech, Budapest, Hungary).

### 2.3. Echocardiography

We performed echocardiography on all patients using Vivid E9 (GE Healthcare, Milwaukee, WI, USA) machine equipped with an M5S-D, 1.5–4.6 MHz transducer, with simultaneous ECG monitoring. For each acquisition, 3 cardiac cycles of uncompressed data were stored in cine-loop format and analyzed without blinding offline by 1 investigator, who was blinded to the clinical characteristics of the patients. All measurements were performed by previously described methods [2].

### 2.4. Two-Dimensional (2D) Echocardiography

Measurement of LA volume from the Simpson method used 4-chamber and apical 2-chamber views at ventricular end-systole, maximum LA size (LAV), and then normalized for body surface area (BSA) as LAV index (LAVi). The apical 2- and 4-chamber views were used to measure left ventricular end-diastolic and end-systolic volumes using the biplane method of disks and EF was calculated. Parasternal long-axis view (2D) was used to measure wall thickness–inter-ventricular septum (IVS) and posterolateral wall (PLW) and the same view was used to measure the standard antero-posterior diameter of the left atrium. Left ventricular mass (LVM) was calculated via area–length method automatically by software according to measures obtained from parasternal cross-sectional view in which mid-ventricular systolic and diastolic epicardial and endocardial surface, with the exclusion of papillary muscles, were traced, as well as the systolic and diastolic mitral-to-apical distance in apical 4-chamber view. LVM = 1, 05 (5/6A2 (L + t)) – (5/6A2L), where A1 is epicardial area at end-diastole (cm^2^), A2 endocardial area at end-diastole (cm^2^), L-ventricular length at end-diastole (cm), t = average wall thickness (cm), 1, 05-specific gravity of the muscle (g/mL). To obtain the indexed value (LVMi) the LVM was divided by BSA.

### 2.5. Doppler Echocardiography

In the apical 4-chamber view, transmitral pulsed-wave (PW) Doppler was obtained at the tips of mitral leaflets, and peak early (E) and late (A) diastolic filling velocities, E/A ratio, and E-wave deceleration time (DTE) were obtained. Tissue Doppler imaging of the mitral annulus was performed at the septal and lateral positions, from which values for the peak early (e′) velocities were obtained and averaged. E/e’ ratio was calculated from E velocity and averaged e’ velocities obtained from septal and lateral positions of the mitral annulus. The maximum tricuspid regurgitation velocity was measured in the apical 4-chamber view at the position of tricuspid annulus using continuous-wave (CW) Doppler.

Strain analysis: For all patients, using EchoPAC Clinical Workstation Software (GE Healthcare, Milwaukee, WI, USA) in post-processing, global left ventricular strain (LVGS) and peak reservoir left atrial strain (LAS) were determined. All images were taken at the frame rate of 60–80/s. Three consecutive cardiac cycles were recorded. Cardiac cycles from three different apical approaches were recorded: 4-chamber view (4CH), 3-chamber view (3CH) and 2- chamber view (2CH). Mitral and aortic valve closure, as observed in the apical 3-chamber view, is used as a recommended end-diastole and end-systole. Endocardial edges were manually marked by the “point-and-click” approach. The epicardial edge was then automatically generated by the system to form a so-called region of interest (ROI). When the ROI was defined, the software automatically divided the same into segments, and the quality score of each segment was automatically calculated, and those segments were classified as acceptable or unacceptable. There was a possibility of manual correction of the edge of each initially unacceptable segment. Those segments that did not have adequate image quality were excluded from the analysis. Left ventricular strain: The LVGS was determined for all 6 left ventricular walls, and the software algorithm automatically segmented the left ventricular walls into 18 sections to form a single bull’s eye model. The mean peak longitudinal strain (LAS-) was calculated as the mid-value of LAS for all segments. The resulting LVGS value was used for further analysis. Left Atrial Strain: A total of 12 segments were analyzed. The software automatically generated longitudinal strain curves for each segment, as well as a curve representing the mean value for all analyzed segments. The first and maximum positive deviation of the strain value was measured at the end of the atrial filling, when the atrium was most stretched (opening of the mitral and closing of the aortic valve)—reservoir strain (LAS). We set the starting point of strain analysis as P-wave.

### 2.6. Statistical Analysis

Continuous variables with normal distribution were presented as mean ± standard deviation and others as median, quartiles, frequencies and percentages. The Student’s *t*-test for independent samples and a Mann–Whitney test were used to compare the mean values of the variables of the examined groups. A *p* value < 0.05 was considered statistically significant. Multivariate regression analysis was used to determine the association between different echocardiographic parameters and DD with elevated LAP independently of age, sex and BMI. The predictive quality of the variables on outcome was evaluated using receiver operating characteristic (ROC) curves. The area under the curve (AUC) was obtained to assess its diagnostic performance, AUC comparison was performed by DeLong’s method. Hosmer and Lemeshow test was used to examine the quality of the obtained combination of echocardiographic parameters in multivariate binary logistic regression. The test showed that the combination was good (*p* = 0.538).

## 3. Results

The basic parameters of the groups are presented in Table 1.

The duration of hypertension was significantly longer in the group with DD with elevated LAP (78.9% vs. 0%, *p* < 0.0001), and there was a significantly higher prevalence of smoking history 47% vs. 25%, *p* = 0.002, respectively. Statistically, significant differences were observed between the majority of examined echocardiographic parameters compared between groups (Table 2).

Univariant and multivariate binary logistic regression was used to examine the influence of examined parameters on the occurrence of DD with elevated LAP. Results are presented in Table 3. Multivariate logistic regression showed that the statistically significant predictors of DD with elevated LAP were LAS (OR 0.834, *p* < 0.0001) and DTE (0.990, *p* = 0.021).

The sensitivity and specificity of different echocardiographic parameters in diagnosing DD with elevated LAP were tested by ROC analysis. Statistically significant predictors of DD with elevated LAP according to the area under the curve were LAS (AUC 0.885 with cut-off 24.27%), E/e’ (AUC 0.879, cut-off 12.35), LVGS (0.800, cut-off −15.9%) and DTE (AUC 0.764, cut-off 142 ms), Table 4.

The set cut-off for LAS > 24.27% was significantly highly prevalent in the group of DD with increased LAP 78.9% when compared to the group without increased LAP 15.4%, *p* < 0.0001. When results of the ROC analysis were compared for chosen parameters in pairs, E/e’ was proven to be a better predictor of DD with elevated LAP than LVGS (*p* = 0.03) and DTE (*p* = 0.006). Peak LAS was a significantly better predictor of DD with elevated LAP when compared with LVGS (*p* = 0.027) and DTE (*p* = 0.004), but no significant difference was observed when compared with E/e’ (*p* = 0.4).

**Figure 1:**
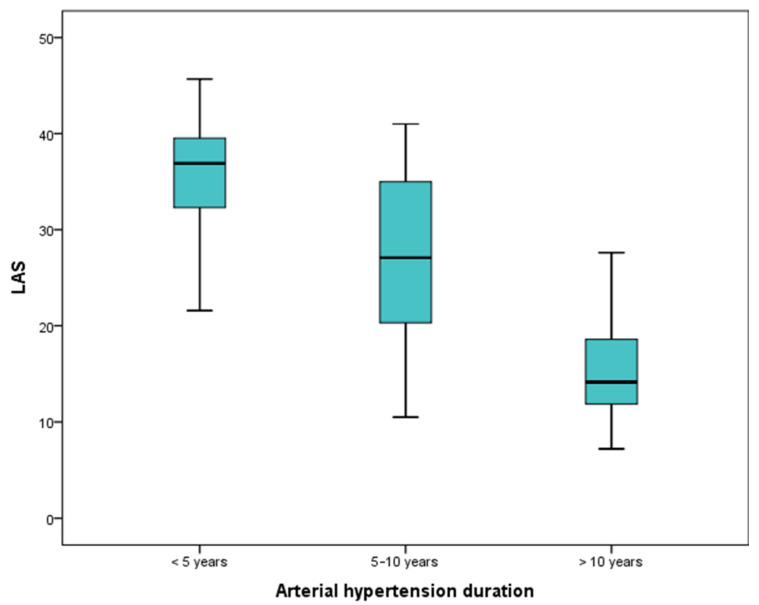
The values of LAS in different arterial hypertension duration.

The values of the LAS significantly decrease with the duration of hypertension, *p* < 0.000 (Figure 1). A set cut-off value of 24.27% is significantly associated with the duration of hypertension (Table 5).

## 4. Discussion

According to our results, we postulate that LAS can be used with a set cut-off <24.27% as a highly sensitive and specific parameter in the population of arterial hypertension for determining DD of advanced stage or to have additional value to confirm or clarify the degree of DD. Only a very few studies have investigated LAS cut-off values and changes in DD in patients with arterial hypertension.

Of all examined echocardiographic parameters, LAS had the highest sensitivity and specificity for diagnosing DD with elevated LAP. Conventional echocardiographic parameters’ correlation with DD grade differs significantly in patients with arterial hypertension. Furthermore, only a few parameters have good sensitivity and specificity that enable them to be used as single predictors of DD with elevated LAP.

Heart failure and cardiovascular disorders represent the leading cause of death in patients with hypertension and diabetes [8]. Abnormalities in DD have been found in early reports in patients with hypertension [9]. Gu et al. found that hypertensive patients with DD exhibit a reduced early phase of systolic dysfunction, which may sustain myocardial contraction, preserving systolic ejection fraction at the expense of impaired diastolic function [10]. Progression of LV DD is related to adverse cardiovascular outcomes [11]. Timely detection of DD in preserved LV systolic function is very important but could be complicated in patients with arterial hypertension [12].

According to data on the prevalence of HF and LV dysfunction in the China Hypertension Survey on 22,158 participants, LV DD was twice more prevalent than LV systolic HF [13]. More than 50% of patients with HF have preserved EF characterized by DD [14]. Noncardiac comorbidities are highly prevalent in HFPEF [15] with hypertension being the most prevalent of all [16]. Hypertension leads to chronic endothelial dysfunction, promoting oxidative stress, inflammation, and atherosclerosis [17,18]. Hyperglycemia’s role in endothelial function impairment is well known together with being a risk factor for severe cardiovascular outcomes, independent of the presence of diabetes [17]. Mutual cardiovascular risk factors play a part in the onset of DD and its progression towards HFpEF, as the incidence of HFpEF increases with rising prevalence of obesity, hypertension, chronic kidney disease, female sex [19] and diabetes [20]. In a study that included 1740 participants, age, female sex, blood pressure, body mass index, serum triglycerides and diabetes were positively associated with worsening diastolic function. Progression of LV DD was also related to the higher prevalence of noncardiac comorbidity and to the incidence of adverse cardiovascular outcomes [11].

The 2013 new paradigm suggested that comorbidities (overweight/obesity, diabetes mellitus, chronic obstructive pulmonary disease, hypertension, anemia and chronic kidney disease) are key factors that initiate a systemic pro-inflammatory state that causes coronary microvascular endothelial inflammation, which contributes to LV stiffness and HFpEF development and progression [21]. This paradigm has been tested in an experimental study with a large (swine) animal model [20]. The co-existence of three common comorbidities (hypertension, hypercholesterolemia and diabetes) led to reduced NO production, impaired coronary artery vasodilatation, myocardial collagen accumulation, reduced capillary/fiber ratio and elevated passive LV stiffness, resulting in increased LV end-diastolic stiffness and a trend towards reduced LV diastolic early-to-late filing velocities, while EF was still preserved [20]. Brandt et al. [22], on a rat animal model, found that obesity negatively affects cardiac output. The presence of oxidative stress and hypertrophic remodeling leads to an elevated E/e’ ratio and mildly reduced ejection fraction. Hypertension in contrast triggered apoptosis, inflammation and fibrosis but did not affect cardiac output and minimally elevated E/e’ ratio.

There has been emerging and growing evidence the usefulness of LAS in the literature. Nevertheless, the data on the hypertension population is scarce. The usefulness of LAS has been proven to have a great prognostic value: in the incidence of atrial fibrillation after aortic valve replacement in patients with non-dilated left atria [23], reduced exercise capacity after myocardial infarction [24], adverse cardiovascular outcomes in patients with mitral regurgitation [25] and in adverse outcomes (total cardiovascular death and heart failure-related hospitalization) in patients with cardiac amyloidosis [26]. Cameli et al. [27] found that in patients with hypertension, early changes occur in peak LAS irrespective of DD, and that E/e’ ratio is the strongest predictor of reduced peak LAS. Our results revealed that the best predictors with mutual connection for the occurrence of DD with elevated LAP were LAS and E/e’ ratio, which is in accordance with the previously mentioned study. Left atrial longitudinal strain strongly correlates with the invasive measurement of LV filling pressure and, therefore, could be easily utilized, in addition to the conventional parameters [28]. In a study on 76 patients who underwent echocardiography and invasive left-heart catheterization, the use of LAS to estimate the LAP was more accurate than the current guidelines [29].

Mondillo et al. found that LAS indices were reduced in hypertensive patients with normal LA size, suggesting that strain abnormalities precede structural LA changes in hypertension [30]. Sahebjam et al., in a similar study on hypertensive patients compared to healthy controls, confirmed these results [31]. Degirmenci et al. showed that LAS reservoir, conduit and booster pump function improved after treatment with renin-angiotensin receptor blockers and beta-blockers for 12 months in patients with mild to moderate hypertension [32]. Hypertension is associated with impaired LA function, as assessed by a strain imaging technique, even before LA enlargement develops and after LV remodeling is accounted for [33]. In our study, we found that the values of LAS strongly correlate with the duration of hypertension.

The LAVi parameter of the left atrial structure is one of the cornerstones of the currently used parameters in diagnosing DD [3]. In our study, LAVi AUC was 0.885 with sensitivity of 75% and specificity of 66.3%. However, LAVi reflects structural changes and recent research suggests that even a left atrium with a normal size can be dysfunctional and that LAVi alone has low sensitivity in the early detection of left atrial DD. Morris et al. [34] postulated that adding LAS to LAVi in the diagnostic algorithm could help increase the detection of LVDD and further stratify indeterminate DD in patients with preserved LVEF. In a study performed on 517 patients with risk factors for DD, LAS had greater sensibility than LAVi in detecting patients with DD as defined by 2016 guidelines, the cut-off of LAS < 23% showed 73% sensitivity and 76% specificity in the identification of DD. These results are very similar to ours. In the presence of normal LAVi, DD was more frequent when a reduction in left atrial strain was present [34]. A recently published study showed that adding LAS as a criterion in the DD assessment significantly reduces the number of indeterminate studies by reclassifying them as normal [35].

A systematic review of 40 meta-analyses (2542 healthy subjects) revealed a normal reference range for reservoir strain of 39% (95% CI, 38–41%) [36]. Only a few studies analyzed LAS changes in different grades of DD in the hypertensive population. However, one study stands out in that it analyzed not only strain changes in different degrees of left ventricular DD in over 200 subjects, but also yielded cut-off values for each degree [28]. Comparing our results with the results of the aforementioned study by Singh et al. in 2016, who performed DD grading according to previously valid 2009 ASE guidelines, we came to the conclusion that the set cut-off values were practically identical for the determination of advanced DD (LAS 24%) with very similar sensitivity and specificity. Frydas et al. [37] in their research showed that LAS could be a useful parameter in the evaluation of DD in patients with heart failure and sinus rhythm, irrespective of LVEF. The set cut-off LAS value for the DD grade II for patients with preserved EF was 21.1 ± 4.8%, which is slightly lower when compared to our results; this could be explained by the different populations of patients with HF and NYHA II-IV. A recently published article on 322 patients with different cardiovascular diseases found that LAS was a good predictor of elevated LV filling pressure and was proposed as a supplementary marker of LV filling pressure [38]. They found that LAS values <18% in patients with reduced EF, were associated with elevated LV filling pressure, and in patients with preserved EF, LAS > 14 was associated with normal LV filling pressure.

Studies related to LVGS in arterial hypertension report that LVGS is less negative in individuals with arterial hypertension [39,40], which fits in with the results obtained in our study. Previous studies have shown that changes in LVGS exist together with changes in DD and that with an increasing degree of DD, LVGS values become worse [39,41]. Singh et al. [28] also set cut-off values of LVGS for each degree of DD and defined that the LVGS value for degree II of DD is −16%, which fits with the cut-off values obtained by our analysis (−15.9%). Singh et al. [28] concluded that, since there are overlapping curves for DD grade I and II, LVGS is not a sufficiently high-quality isolated indicator of DD. In our study, we showed that LVGS can be used as a predictor of DD with good sensitivity, but significantly less specificity than LAS, with a significantly smaller area of the ROC curve, and in such a way it is not a reliable stand-alone marker of DD with elevated LAP.

### Limitations

We acknowledge that the limitations of the study are: Firstly, there was a small number of patients, and it was a one-center study; therefore, the data may be geographically and ethnically influenced. Secondly, the measurements are performed only noninvasively and not invasively. Invasive measurement is the gold standard for intracardiac pressure measurements but are difficult to achieve in everyday clinical practice due to numerous reasons; future studies could confirm the results of noninvasively measured parameters. Thirdly, the calculation of LAS is not very simple and not performed routinely in everyday practice, and it is still costly due to non-standard software, but in future years, with the advancement of technology, these difficulties can be overcome. Fourthly, the lack of data on follow-up or biomarkers such as natriuretic peptides. These data realized in future studies could provide significant value about LAS as a predictor of heart failure or adverse cardiovascular outcomes in a population of patients with arterial hypertension.

## 5. Conclusions

In conclusion, the results of our study indicate that a set cut-off value below 24.27% for left atrial strain is a valuable, highly sensitive and specific marker of diastolic dysfunction that could be used in everyday practice to facilitate diastolic function assessments and easily reveal patients with diastolic dysfunction with increased left atrial pressure that is a risk factor and precedes heart failure in the population of patients with arterial hypertension. Our study and other larger studies can add additional value to include LAS in DD assessment in future clinical practice guidelines.

## Figures and Tables

**Table 1:** Characteristics of the patients with arterial hypertension.

Parameter	Group with Normal LAP *N* = 104 (57.77%) Mean ± SD or %	Group with Elevated LAP *N* = 76 (42.22%) Mean ± SD or %	*p*
Age (years)	53.11 ± 6.15	54.95 ± 7.2	0.07
Gender (f)	55.4%	44.6%	0.1
BMI (kg/m^2^)	28.32 ± 3.58	28.93 ± 4.14	0.2
History of hypertension ≤ 5 years	58 (55.8%)	2 (2.6%)	<0.0001
History of hypertension 5–10 years	46 (44.2%)	14 (18.4%)	0.0003
History of hypertension ≥ 10 years	0	60 (78.9%)	<0.0001
SBP (mmHg)	126.2 ± 12.4	128 ± 15.4	0.33
DBP (mmHg)	72 ± 10.2	75 ± 13.3	0.08
HR (bpm)	75 ± 20.1	78 ± 25.4	0.37
History of diabetes	23 (22.1%)	30 (39.4%)	0.01
Smoking history	26 (25%)	36 (47%)	0.002
History of hyperlipidemia	20 (19.2%)	28 (36.8%)	0.008
Number of antihypertensive agents/day ≤ 2	58 (55%)	46 (44%)	0.1
Number of antihypertensive agents/day > 2	36 (47%)	40 (52%)	0.5
Users of β blockers	28 (26.9%)	22 (28.9%)	0.7
Users of ACE/ARB	52 (50%)	47 (61.8%)	0.1
Users of CCB	34 (32.7%)	32 (42.1%)	0.1
Users of Thiazides	36 (34.6%)	30 (39.5%)	0.5

Legend: ACE—angiotensin-converting enzyme (ACE) inhibitors, ARBs—angiotensin receptor blockers, BMI—body mass index, CCB—calcium channel blocker, DBP—diastolic blood pressure, HR—heart rate, LAP—left atrial pressure, SBP—systolic blood pressure.

**Table 2:** Echocardiographic parameters of the study population.

Parameter	Group with Normal LAP *N* = 104 (57.77%) Mean ± SD or %	Group with Elevated LAP *N* = 76 (42.22%) Mean ± SD or %	*p*
IVS/PLW (cm)	1.17 ± 0.12	1.27 ± 0.14	<0.0001
LVDd (cm)	4.7 (4.3; 5.1)	4.7 (4.4; 5.1)	0.38
LVDs (cm)	2.9 (2.6; 3.2)	2.9 (2.7; 3.3)	0.19
EF (%)	63 (60; 63)	61 (59; 63)	0.007
LVMi (g/m^2^)	90.46 ± 15.42	113.32 ± 29.99	<0.0001
Vp (cm/s)	52.87 ± 7.14	47.3 ± 4.52	<0.0001
E/e’ ratio	10.42 ± 2.23	15.13 ± 3.86	<0.0001
IVRT (mm/s)	85.48 ± 12.54	71.07 ± 18.01	<0.0001
LA (cm)	3.7 (3.4–3.9)	4.3 (3.9–4.6)	<0.0001
LAVi (mL/m^2^)	34 (28.61–46.17)	35.80 (41.93–57.05)	<0.0001
LVM (g)	182.2 (154.1–204)	215.03 (196.75–250)	<0.0001
E-wave (m/s)	0.7 (0.6–0.8)	0.9 (0.7–1.0)	<0.0001
E/A ratio	0.94 (0.75–1.19)	1.19 (0.83–1.4)	0.002
DTE (ms)	163 (141–185)	132.5 (121.5–143)	<0.0001
e’ (m/s)	0.06 (0.05–0.08)	0.05 (0.05–0.06)	<0.0001
LVGS (%)	−17.62 (−19.35–15.36)	−14.58 (−15.5–−13.39)	<0.0001
LAS (%)	34.75 (27.16–38.45)	15.84 (12.22–21.35)	<0.0001
RVDd (cm)	2.4 (2.2; 2.6)	2.4 (2.1; 2.6)	0.93
TR Vmax (m/s)	1.95 (0–2.48)	2.1 (1.05–2.75)	0.04
TAPSE (mm)	24.6 ± 3.2	23.8 ± 4.6	0.1
RVs’ (cm/s)	14 ± 2.1	13.6 ±1.9	0.1
MR mild	14 (35.9%)	25 (64.1%)	0.003
TR mild	12 (33.3%)	24 (66.7%)	0.001

Legend: DTE—deceleration time, EF—ejection fraction, IVRT—isovolumic relaxation time, IVS—inter-ventricular septum thickness, LA—left atrial parasternal long-axis diameter, LVDd—left ventricle diameter diastolic, LVDs—left ventricle diameter systolic, LVGS—longitudinal left ventricular strain, LVM—left ventricular mass, LVMi—LVM index, LAS—peak left atrial strain, LAVi—LAV index, MR—mitral regurgitation, PLW—posterolateral wall thickness, RVDd—right ventricle diastolic diameter, TR—tricuspid regurgitation, TR Vmax—The maximal tricuspid regurgitation velocity, Vp—velocity of propagation.

**Table 3:** Univariant and Multivariate binary logistic regression for selected echocardiographic parameters.

Parameter	Univariant		Multivariant	
	Odds Ratio (95% CI)	*p* Value	Odds Ratio (95% CI)	*p* Value
LVMi (g/m^2^)	1.063 (1.038–1.088)	<0.0005	/	ns
LVGS %	1.325 (1.169–1.503)	<0.0005	/	ns
Vp (cm/s)	0.825 (0.766–0.889)	<0.0005	/	ns
DTE (ms)	0.984 (0.976–0.993)	<0.0005	0.990 (0.981–0.998)	0.021
LAS %	0.830 (0.790–0.872)	<0.0005	0.834 (0.793–0.876)	0.000

Table legend: DTE—deceleration time, LAVi—LAV index, LAS—peak left atrial strain, LVMi—left ventricular mass index, LVGS—longitudinal left ventricular strain, TR Vmax—The maximal tricuspid regurgitation velocity, Vp—velocity of propagation.

**Table 4:** Analysis of ROC curves for selected echocardiographic parameters.

Parameter	AUC	Std. Error	*p* Value	Cut-off	Sensitivity (%)	Specificity (%)
E/e’	0.879	0.027	<0.0005	12.35	82.9	77.9
LAVi (mL/m^2^)	0.689	0.040	<0.0005	39.90	75.0	66.3
LVMi (g/m^2^)	0.763	0.035	<0.0005	97.33	76.3	66.3
GLS (%)	0.800	0.033	<0.0005	−15.90	85.5	70.2
TR V max (m/s)	0.587	0.043	=0.046	2.47	40.8	75.0
Vp (cm/s)	0.761	0.035	<0.0005	50.50	84.2	65.4
DTE (ms)	0.764	0.040	<0.0005	142.00	75.0	74.0
LAS (%)	0.885	0.025	<0.0005	24.27	78.9	84.6

Table legend: LAVi—left atrial volume index, LVMi—left ventricular mass index, GLS—longitudinal left ventricular strain, TR Vmax—The maximal tricuspid regurgitation velocity, Vp—velocity of propagation, DTE—deceleration time, LAS—peak left atrial strain, AUC—area under curve.

**Table 5:** LAS values in different hypertension durations.

Parameter	History of Hypertension ≤ 5 Years	History of Hypertension 5–10 Years	History of Hypertension ≥ 10 Years	*p*
LAS %	36.9 (32.2; 41.2)	27 (20.5; 35)	14.1 (11.8; 19.4)	0.000
LAS ≤ 24.27	59 (98.3%)	38 (63.3%)	1 (11.7%)	0.000

## Data Availability

The data presented in this study are available on request from the corresponding author.
